# Long‐term adverse cardiovascular changes induced by cocoa shell extract administration through lactation in fetal undernourished and control rats

**DOI:** 10.1113/EP093742

**Published:** 2026-07-02

**Authors:** Santiago Ruvira, Pilar Rodríguez‐Rodríguez, Metee Iampanichakul, Begoña Quintana‐Villamandos, Dolores Morales, David Ramiro‐Cortijo, Silvia M. Arribas

**Affiliations:** ^1^ Department of Physiology, Faculty of Medicine Universidad Autónoma de Madrid Madrid Spain; ^2^ Food, Oxidative Stress and Cardiovascular Health (FOSCH) Research Group Universidad Autónoma de Madrid Madrid Spain; ^3^ Hospital La Paz Institute for Health Research (IdiPAZ) Hospital Universitario La Paz Madrid Spain; ^4^ Department of Physiology Khon Kaen University Khon Kaen Thailand; ^5^ Department of Anesthesiology Hospital General Universitario Gregorio Marañón Madrid Spain; ^6^ Department of Pharmacology and Toxicology Universidad Complutense de Madrid Madrid Spain; ^7^ Confocal Microscopy Service (SiDI), Faculty of Medicine Universidad Autonoma de Madrid Madrid Spain

**Keywords:** cocoa shell extract, ejection fraction, lactation, mesenteric resistance artery, remodelling, vasodilatation

## Abstract

Cocoa shell extract (CSE; rich in antioxidant compounds) administered to adult hypertensive rats exposed to fetal undernutrition (MUN) reduces blood pressure, improving cardiovascular alterations. We aimed to explore lactation as a reprogramming window, evaluating the long‐term effects of CSE supplementation during this period. MUN and control (C) dams were given 250 mg/kg/day CSE (5 days/week) or vehicle (VEH) in gelatine throughout lactation. Blood pressure (tail‐cuff plethysmography), heart (echocardiography) and mesenteric resistance artery (MRA) function (wire myography) and structure (pressure myography, confocal microscopy) were studied in 8‐month‐old offspring. MUN‐VEH rats had significantly higher blood pressure and reduced MRA internal diameter compared with C‐VEH counterparts. MUN‐VEH males also had smaller left ventricular mass index, acetylcholine, sodium nitroprusside relaxations and increased stiffness, whereas MUN‐VEH females had elevated maximal noradrenaline‐evoked contractions. CSE administration led to lower body weight in most groups and a lower blood pressure in MUN‐CSE, but not C‐CSE rats, compared with non‐treated counterparts. Supplementation reduced left ventricular mass index, heart rate and ejection fraction in all groups and worsened MRA acetylcholine‐mediated vasodilatation in MUN‐CSE females and C‐CSE males and females. CSE induced medial layer hypertrophy in all groups; it increased internal diameter only in MUN‐CSE rats and it elevated wall‐to‐lumen ratio in C‐CSE rats. In conclusion, although CSE supplementation during lactation prevented the development of hypertension in MUN rats, the induced cardiovascular functional and structural alterations, particularly in control offspring, suggest detrimental effects of CSE bioactive components, at least at the dose used. This study highlights the need for caution when administering supplements during lactation.

## Abstract (highlights)

- **What is the central question of this study?**
  Can supplementation during lactation with cocoa shell extract (CSE; rich in antioxidant bioactive compounds) prevent the development of hypertension and cardiovascular alterations induced by fetal undernutrition? What are its effects in control rats?
- **What is the main finding and its importance?**
  Overall, CSE administered during lactation did not confer benefits, in contrast to the effects reported in adulthood. Although it prevented the development of hypertension in rats exposed to fetal undernutrition, the observed reduction in ejection fraction and impaired small‐artery vasodilatation across all groups suggest potential adverse effects of CSE bioactive compounds when administered during this sensitive life period.

## 1 INTRODUCTION

Exposure to stress factors during the fetal and postnatal periods increases the risk of developing cardiometabolic diseases, a process known as developmental origins of health and disease (DOHaD) or cardiometabolic programming. Exposure of experimental animals to early‐life insults has been instrumental in elucidating the mechanisms underlying this association, providing evidence that oxidative stress is one of the major contributing factors (Hsu & Tain, 2021; Morton et al., 2016; Rodríguez‐Rodríguez et al., 2018). In a rat model of global maternal nutrient restriction during gestation (MUN model), we have characterized cardiovascular alterations across the lifespan and demonstrated the crucial role of early antioxidant deficiency as a key factor for the development of hypertension and cardiovascular structural and functional alterations (Rodríguez‐Rodríguez et al., 2015, 2017, 2023).

Given the pivotal implication of oxidative stress in cardiovascular disease programming, antioxidant therapy has been proposed as an intervention strategy. We have supplemented adult hypertensive MUN rats with cocoa shell extract (CSE), a by‐product from the chocolate industry enriched in methylxanthines and polyphenols with antioxidant properties (Cañas et al., 2023, 2024), and demonstrated its capacity to reduce blood pressure, improving resistance artery vasodilatation (Ruvira et al., 2024) and elasticity (Rodríguez‐Rodríguez et al., 2025), at least in part, by decreasing vascular reactive oxygen species.

Within the framework of the DOHaD paradigm, the concept of reprogramming has recently attracted considerable attention, supporting a shift in therapeutic strategies from adulthood to the perinatal period, with the aim of preventing disease onset (Silva et al., 2019; Tain & Hsu, 2024). Interventions with antioxidant therapy during gestation have demonstrated dual effects regarding effectiveness to prevent long‐term cardiometabolic alterations in several DOHaD animal models. Positive results have been found with *N*‐acetylcysteine (Herrera et al., 2017; Yonatan et al., 2025), short‐chain fatty acids (Hsu et al., 2025), melatonin (Chitimus et al., 2020), epigallocatechin gallate from green tea, curcumin, quercetin (Fortunato et al., 2021), resveratrol (Hsu et al., 2025) or cocoa polyphenols (Mariné‐Casadó et al., 2022). However, there is also evidence of negative effects of supplementation during gestation with green tea (Hachul et al., 2018a, 2018 *b*), epigallocatechin (Lewicki et al., 2017), grape procyanidins (del Bas et al., 2015) or naringin (Gindri dos Santos et al., 2021), sometimes affecting control offspring. These data suggest the need for additional studies to assess the effectiveness and safety of interventions in early life with food‐derived antioxidants.

Lactation is another key developmental window with high plasticity, and thus capacity for reprogramming. In fact, in the MUN rat model of fetal undernutrition, we have established the significant role of this period in shaping cardiometabolic alterations initiated during fetal life (Monedero Cobeta et al., 2024; Rodríguez‐Rodríguez et al., 2022a). These findings support the notion that lactation might represent a valuable window for intervention within the DOHaD framework. The limited evidence available underlines the need for studies specifically evaluating supplementation specifically during this period (without an intervention in gestation) in models of fetal programming. Such research is essential to advance our understanding and facilitate clinical translation.

Based on the effectiveness of CSE administration during adulthood to reverse cardiovascular alterations induced by fetal undernutrition in MUN model, in the present study we hypothesised that supplementation through lactation might reprogram the phenotype. Given the controversy of supplementation during pregnancy, we also aimed to assess the impact of administration of CSE during lactation on non‐programmed control offspring, evaluating blood pressure, cardiac function and resistance vasculature in adulthood.

## 2 MATERIALS AND METHODS

### 2.1 Ethical approval

Experimental procedures were conducted in accordance with the Spanish legislation (RD 53/2013), Directive 2010/63/EU on the protection of animals used for scientific purposes, and the *Guidelines for the Care and Use of Laboratory Animals*. The experiments were conducted in adult Sprague–Dawley rats obtained from the Animal House facility of the Universidad Autónoma de Madrid (ES‐28079‐0000097). All procedures were approved by the Animal Welfare Committee of Universidad Autónoma de Madrid (CEI‐UAM 96‐1776‐A286) and by the Regional Environment Committee of the Comunidad Autónoma de Madrid (RD 53/2013; Ref. PROEX 04/19). The experiments also conform to the principles, regulations and standards for reporting animal experiments (Grundy, 2015).

### 2.2 Animal model and experimental design

Sprague–Dawley rats were housed in buckets of appropriate size according to their age and sex, on aspen wood bedding and were kept on a 12 h–12 h light–dark photoperiod, at a constant temperature of 22°C and 40% relative humidity. The rats were fed with a standard breeding diet (Euro Rodent Diet 22; 5LF5, Labdiet, Madrid, Spain; 55% carbohydrate, 22% protein, 4.4% fat, 4.1% fibre and 5.4% mineral), and water was provided ad libitum. Animal health and welfare were monitored regularly by qualified Animal House Facility staff.

**FIGURE 1:**
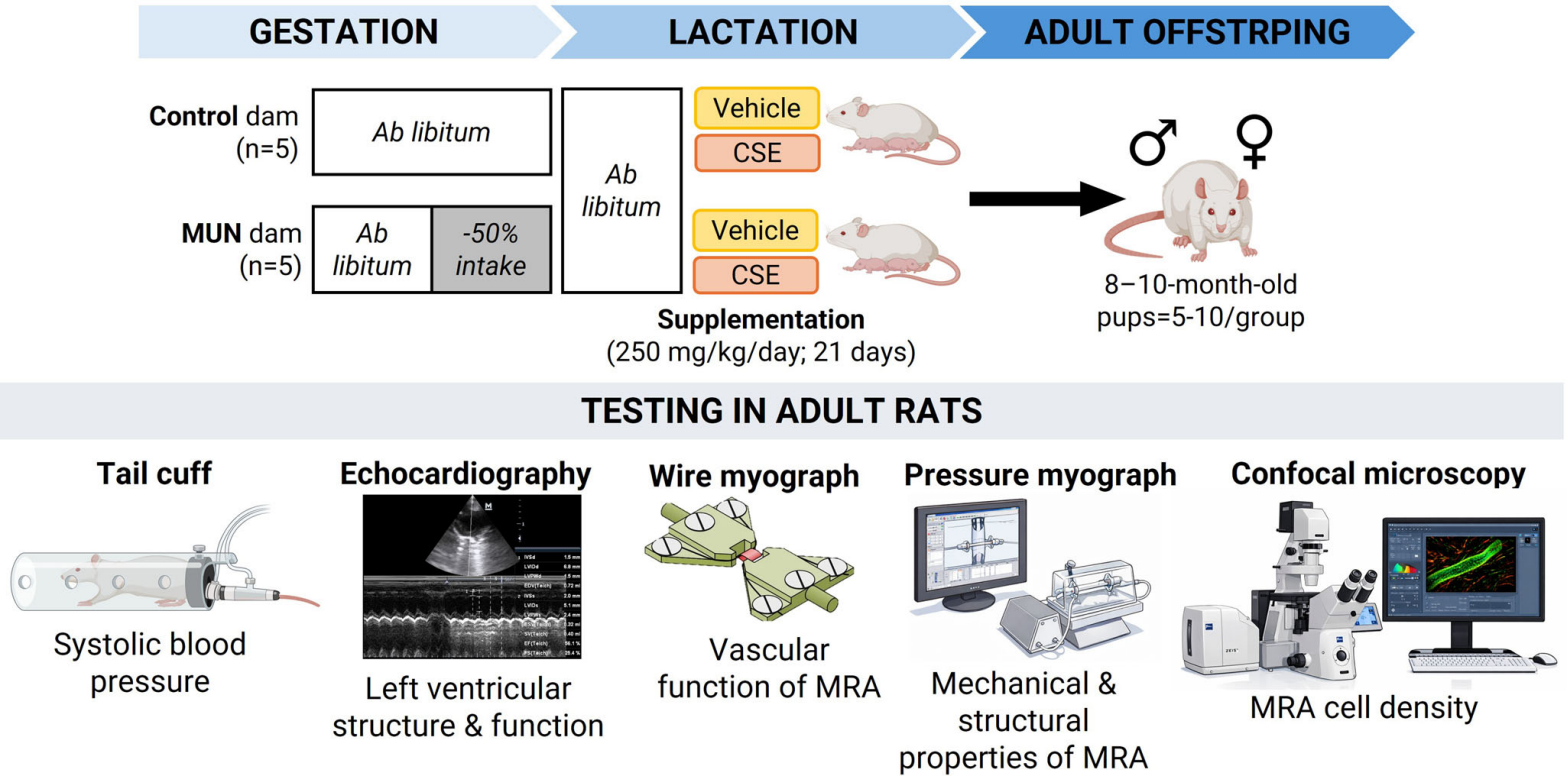
Experimental design. Abbreviations: CSE, cocoa shell extract; MRA, mesenteric resistance artery; MUN, maternal undernutrition.

The model of hypertension programming was developed by maternal undernutrition during the second part of gestation (MUN model), as previously described (Ruvira et al., 2024). Briefly, after mating, day 1 of gestation was established by the presence of a vaginal plug in the cage. The dams were then randomly allocated to Control (C) or MUN groups. Four experimental groups were established: control dams supplemented with vehicle (C‐VEH, *n* = 5), control dams supplemented with CSE (C‐CSE, *n* = 5), MUN rats supplemented with vehicle (MUN‐VEH, *n* = 5) and MUN rats supplemented with CSE (MUN‐CSE, *n* = 5). Control dams received food ad libitum throughout gestation, whereas MUN rats were fed ad libitum during the first 10 days, then with 12 g/day (50% of the usual rat daily intake). After delivery, the pups were kept with the same mother during the lactation period, standardizing the litter in 12 individuals, 6 females and 6 males whenever possible. From birth, both control and MUN dams received food ad libitum and were supplemented either with vehicle (VEH) or with CSE (detailed in section 2.3. supplementation procedure). At weaning (established at 21 days), male and female offspring were separated into different cages and maintained on standard diet and water (both provided ad libitum) until the age of 8–10 months of age, when the following experimental procedures were conducted in one or two males and one or two females from each litter: (1) echocardiography; (2) blood pressure; (3) animal euthanasia and mesenteric resistance artery (MRA) dissection; (4) MRA function experiments; (5) MRA structure experiments; and (6) confocal microscopy experiments. Figure 1 shows the experimental design.

### 2.3 CSE supplementation procedure

Supplementation was based on voluntary acceptance of a gelatine cube, as previously described (Ruvira et al., 2023). The vehicle was a neutral gelatine (Inkafoods, S.L., Barcelona, Spain) dissolved in hot water at a concentration of 140 g/L and placed in a mould to form 1 cm^3^ cubes and was allowed to solidify at room temperature. CSE was obtained from cocoa shell, provided by Chocolates Santocildes S.A. (https://www.chocolatessantocildes.com; León, Spain) through optimized aqueous extraction (Rebollo‐Hernanz et al., 2021). The composition of CSE has been characterized previously as rich in phenolic compounds, mainly hydroxybenzoic acids (gallic and protocatechuic) and flavan‐3‐ols (catechin and epicatechin) and methylxanthines, particularly theobromine and caffeine. To prepare the cubes containing CSE, the extract was incorporated into the vehicle gelatine prior to solidification. The exact composition can be found in the paper by Braojos et al. (2023).

CSE supplementation was conducted in the dams throughout the lactation period (3 weeks) at a dose of 250 mg/kg/day for 5 days/week. This dose was used because we have demonstrated previously that it ensures the presence of the main bioactive compounds in rat plasma (Ramiro‐Cortijo et al., 2025) and is also effective in the reduction of blood pressure when given to adult MUN rats for 3 weeks (Ruvira et al., 2024). To ensure the correct dosage (250 mg/kg), the CSE cube size was adjusted according to the weight of the rat, which was monitored weekly. To avoid rejection, prior to mating all the rats underwent a 3‐ to 5‐day training period with vehicle, until they accepted and ate the entire cube. We have previously demonstrated that the rats remember this protocol for 2 months (Ruvira et al., 2023). No rejection of cubes with either CSE or VEH was detected in the present study. No alterations in maternal behaviour were detected during the supplementation period with either CSE or VEH, with dams displaying typical pup care.

### 2.4 Systolic blood pressure measurements

Systolic blood pressure (SBP) was assessed in the offspring non‐invasively by tail‐cuff plethysmography coupled with a pressure acquisition system (CIBERTEC Niprem 645, Madrid, Spain), as previously described (Gutiérrez‐Arzapalo et al., 2020). Rats were initially warmed at 37°C for 10 min to induce vasodilatation, and blood pressure was measured with the rats in a warm, dark soft piece of cloth to reduce stress. A pressure cuff was placed at the base of the tail, and several measurements were taken during 3 days consecutively. The first day was used for rat habituation, and only the data obtained during the last 2 days were used for statistical analysis.

### 2.5 Transthoracic echocardiography

Transthoracic echocardiography was performed in the offspring as previously described (Monedero Cobeta et al., 2024), with rats under general anaesthesia with 80 mg/kg ketamine hydrochloride (AuroMedics Pharma LLC, Ireland) and 10 mg/kg diazepam (Hospira, Inc., IL, USA). Transthoracic echocardiography was performed using the VIVID q‐system (GE Healthcare, Germany) equipped with a 13 MHz probe (12S‐RS, GE Healthcare, Germany), with the animals in left lateral decubitus, using M‐mode imaging of the parasternal short‐axis (papillary‐level) view. Values were determined by averaging the measurements of three consecutive cardiac cycles in accordance with the American Society of Echocardiography guidelines. The above measurements were used to calculate heart rate (HR) and left ventricular mass (LVM) using the equation below, where LVIDd is left ventricular end‐diastolic internal diameter; LVIDs is the left ventricular end‐systolic internal diameter; IVDs is the interventricular septum thickness at diastole; and PWd is posterior wall thickness at diastole. LVM was divided by rat body weight to obtain left ventricular mass index (LVMI).

$$\mathrm{LVM}=0.8[1.04\times {\left(\mathrm{IVSd}+\mathrm{LVIDd}+\mathrm{PWd}\right)}^{3}-\mathrm{LVID}d^{3}]+0.6$$

### 2.6 MRA isolation and wire myography

The rats were euthanized by CO_2_ inhalation followed by cardiac exsanguination. The mesenteric bed was isolated and placed on Krebs–Henseleit solution (KHS) of the following composition (mM): NaCl, 115; KCl, 4.6; MgSO_4_, 1.2; KH_2_PO_4_, 1.2; NaHCO_3_, 25.0; CaCl_2_, 2.5; EDTA, 0.01; and glucose, 11. Initially, branches of the superior mesenteric resistance artery were dissected in cold KHS, removing fat and connective tissue. Some branches were stored for the study of vascular structure and mechanical function using pressure myography (see section 2.7), and the remaining branches were used for wire myography to analyse vascular function.

Two‐millimetre‐long sections were mounted in a four‐chamber wire myograph system (Multi Wire Myograph System 610M, DMT, Denmark), coupled to a Powerlab data acquisition system (AD Instruments, Castle Hill, NSW, Australia). The MRA was mounted using 25 µm tungsten wires in KHS at 37°C and gassed with a 5% CO_2_ and 95% O_2_ mixture. After a resting period of 15 min, with the wires touching each other, MRAs were gradually stretched in periods of 1 min until achieving a standard tension of ∼4 mN (equivalent to ∼50 mmHg of transmural pressure), according to standard protocols (Haam et al., 2022; Herrera et al., 2010; Mulvany & Halpern, 1977). After a 30 min equilibration period, a 125 mM KCl solution was used for testing the integrity of the arteries, followed by three washes with KHS until reaching the baseline force generation. Concentration–response curves were performed with the following drugs and solutions: high‐potassium KHS (10.72–125 M) and noradrenaline (NA; 10^−8^–10^−4^ M), with three consecutive KHS washing periods between them until the vessels reached the baseline. Thereafter, the vessels were precontracted with 10^−5^ M serotonin, and concentration–response curves to acetylcholine (ACh; 10^−10^–10^−4^ M) and sodium nitroprusside (SNP; 10^−10^–10^−4^ M) were tested with three consecutive KHS washing periods between them. Responses to noradrenaline were calculated as a percentage of KCl contraction, and responses to vasodilators were calculated as a percentage of their respective serotonin precontraction. For statistical analysis, the area under the curve (AUC), maximal response and pD_2_ values were calculated.

### 2.7 Pressure myography

Mechanical and structural properties of the MRA were evaluated with a pressure myograph (Danish Myo Tech, Model P100, J.P. Trading I/S, Aarhus, Denmark). The MRA segment was placed in 0‐Ca^2+^ KHS (omitting calcium and adding 10 mM EGTA) in an organ bath and mounted between two glass cannulae secured with nylon suture. The organ bath was located on the stage of an inverted microscope (Zeiss Axiovert, Braun; Germany) coupled to a charge‐coupled device camera (Sony XC‐73CE, monochrome, Bangkok, Thailand) and a ×10 objective. Following a 15 min stabilization period at a pressure of 80 mmHg and temperature of 37°C, intraluminal pressure was set to 5 mmHg (the minimum pressure to avoid vessel collapse, which was considered zero pressure), and the arterial segment was exposed to increasing intraluminal pressures in steps of 20 mmHg until a maximum of 140 mmHg, taking images at each pressure after a resting period of 3 min between pressure steps. Finally, pressure was adjusted at 80 mmHg, artery segments were fixed in 4% paraformaldehyde for 1 h and stored in a refrigerator for confocal microscopy experiments.

Images were analysed with ImageJ software (https://imagej.net/software/fiji/), measuring internal diameter (Di; lumen) and external diameter (De). From these data, wall thickness (WT), cross‐sectional area (CSA) and wall‐to‐lumen ratio (W/L) were calculated using the following equations, as previously described (Gutiérrez‐Arzapalo et al., 2020):

$$\mathrm{WT}=\frac{\mathrm{De}-\mathrm{Di}}{2};\mathrm{CSA}=\frac{\pi }{4}\times \left(De^{2}-Di^{2}\right);\mathrm{and}W/L=\frac{\mathrm{De}\;-\;\mathrm{Di}}{2\mathrm{Di}}$$

Incremental Young's elastic modulus (*E*_inc_) was used to evaluate the vascular elasticity. *E*_inc_ was assessed by fitting the stress (σ)–strain (ԑ) data from each MRA segment to an exponential curve: $\sigma =\sigma_{\mathrm{origin}}^{\beta \varepsilon }$

The β‐values are directly proportional to *E*_inc_ and, thus, can be used as a measure of the degree of elasticity (the higher the β‐value, the higher the stiffness of the artery).

$$\mathrm{Circumferential}\;\mathrm{wall}\;\mathrm{stress}\;\left(\sigma \right)=\frac{P_{x}\times \mathrm{Di}}{2\mathrm{WT}}$$

where *P*_x_ is the intraluminal pressure in newtons per metre squared (1 mmHg = 133.4 N/m^2^) and WT is the wall thickness at each distending pressure.

$$\mathrm{Circumferential}\;\mathrm{wall}\;\mathrm{strain}\;\left(\varepsilon \right)=\frac{\mathrm{Di}-D_{0}}{D_{0}}$$

where Di is the internal diameter at each pressure and *D*_0_ is the diameter at 5 mmHg.

### 2.8 Confocal microscopy

The pressure‐fixed segments were initially washed in saline solution, then incubated with the nuclear dye DAPI at 1:500 (from stock solution, Invitrogen, Thermo Fisher, Madrid, Spain), at room temperature for 20 min in the dark. Thereafter, they were washed twice with saline solution for 20 min each at room temperature in the dark. Finally, the segment was mounted on a slide provided with a small well made of silicon spacers filled with antifading agent CITIFLUOR‐AF (Life Technologies, Carlsbad, CA, USA) and covered with a coverglass. Segments were visualized with a Leica SP5 spectral confocal microscope (Leica Microsystems, Germany) at 405 nm excitation/410–475 nm emission wavelengths, with a ×40 oil immersion objective. From each MRA segment, two stacks of 1‐µm‐thick serial optical sections were obtained throughout the arterial wall in two different regions with zoom × 2, as previously described (Gutiérrez‐Arzapalo et al., 2020).

Quantification was performed with FIJI free software. The first image of the stack was established as the image with the first visible adventitial cell nuclei, and the last was the image with the last visible vascular smooth muscle cell (VSMC) nuclei, considering the shape and orientation of the nuclei, which allow adventitial and VSMCs to be distinguished clearly. From each stack of images, the numbers of adventitial and VSMCs were counted, layer thickness was determined by the number of planes showing each cell type, and layer volume was calculated as layer thickness × image area. From these data, adventitia and medial layer cell densities were calculated (number of cells/layer volume). The average of the two stacks of images from the same segment was used for statistical analysis.

### 2.9 Statistical analysis

Statistical analysis was performed with R software (v.4.5.2, 2026, R Core Team, Vienna, Austria) within the RStudio interface (2026.01.0; IDE; https://posit.co) using *rio, dplyr, compareGroups, ggpattern, ggpubr, devtools, openxlsx* and *ggplot2* packages. The sample size (*n*) values represent a single rat. Data were analysed including outliers and separately by sex. The data were represented as box‐and‐whisker plots by median and interquartile range [Q1; Q3], and the Mann–Whitney *U*‐test was applied to test the difference in ranks between groups. Additionally, a linear regression model was used to evaluate interaction (supplementation × group), extracting the β‐coefficient ± SE and *P*‐value. A value of *P *< 0.05 was considered significant.

## 3 RESULTS

### 3.1 Body weight and blood pressure

Body weight was significantly lower in MUN‐VEH compared with C‐VEH rats, both in males and in females. Control male and female rats supplemented with CSE (C‐CSE) had significantly lower body weight compared with C‐VEH sex‐matched counterparts. MUN‐CSE males also had significantly lower body weight compared with MUN‐VEH males. However, MUN‐CSE females did not show significant differences compared with MUN‐VEH females (Table 1). A significant interaction between supplementation × group was detected for females, but not for males (Table 2).

**TABLE 1:** Body weight and systolic blood pressure.

Parameter	Control		*P* ^1^	MUN		*P* ^2^	*P* ^model^
**Male**	VEH (*n* = 10)	CSE (*n* = 10)		VEH (*n* = 10)	CSE (*n* = 10)		
Weight, g	510 [499; 527]	471 [432; 492]	**0.007**	460 [441; 479]	430 [412; 444]	**0.041**	**0.001**
SBP, mmHg	130 [128; 141]	139 [135; 143]	0.118	155 [149; 163]	139 [135; 145]	**0.006**	**0.001**
**Female**	VEH (*n* = 10)	CSE (*n* = 9)		VEH (*n* = 9)	CSE (*n* = 9)		
Weight, g	334 [316; 355]	277 [274; 293]	**0.001**	285 [282; 300]	282 [282; 290]	0.564	**0.011**
SBP, mmHg	128 [119; 132]	132 [127; 135]	0.159	135 [132; 151]	127 [126; 135]	**0.024**	**0.009**

*Note*: Data show the median and interquartile range [Q1; Q3]. The *P*‐value was extracted from the Mann–Whitney *U*‐test, with *P* ^1^ being control‐VEH vs. control‐CSE; P^2^, MUN‐VEH vs. MUN‐CSE; and *P* ^model^, control vs. MUN vehicle (VEH) groups. Significant differences are highlighted in bold; *n* indicates the number of animals.

Abbreviations: CSE, cocoa shell extract; MUN, maternal undernutrition; SBP, systolic blood pressure; VEH, vehicle.

**TABLE 2:** Linear regression model with interaction effect.

Parameter	Male		Female	
	β ± SE	*P*‐value	β ± SE	*P*‐value
Body weight	20.67 ± 19.30	0.307	37.88 ± 13.87	**0.010**
Systolic blood pressure	−21.29 ± 5.95	**0.001**	−17.74 ± 5.47	**0.003**
Left ventricular internal diameter at diastole	0.61 ± 0.38	0.125	−0.05 ± 0.34	0.867
Left ventricular internal diameter at systole	0.01 ± 0.39	0.988	−0.38 ± 0.37	0.316
Left ventricular mass index	0.25 ± 0.10	**0.019**	−0.05 ± 0.06	0.370
Left ventricular ejection fraction	3.48 ± 5.02	0.496	7.50 ± 5.38	0.180
Shortening fraction	3.39 ± 6.57	0.611	6.90 ± 6.76	0.321
Heart rate	−31.23 ± 20.30	0.140	−15.33 ± 22.84	0.510
K^+^ contraction	1.34 ± 3.77	0.724	−0.45 ± 3.10	0.886
Maximum noradrenaline contraction	−5.54 ± 4.79	0.257	−13.84 ± 6.79	0.053
Maximum acetylcholine relaxation	46.11 ± 9.70	**<0.001**	7.24 ± 5.71	0.220
Maximum sodium nitroprusside relaxation	1.14 ± 12.0	0.925	9.74 ± 9.10	0.300
Internal diameter	0.99 ± 0.38	**0.013**	1.33 ± 0.52	**0.015**
Cross‐sectional area	0.30 ± 0.13	**0.031**	0.12 ± 0.10	0.250
Wall‐to‐lumen ratio	0.51 ± 4.63	0.913	−3.16 ± 3.13	0.320
Beta value	−1.68 ± 1.17	0.161	−0.80 ± 1.13	0.485
Adventitial thickness	−4.92 ± 3.34	0.159	2.58 ± 2.64	0.344
Cell density adventitial	−0.10 ± 0.71	0.892	−0.07 ± 0.91	0.939
Medial thickness	5.54 ± 5.65	0.341	0.15 ± 5.29	0.978
Vascular smooth muscle cell density	−0.13 ± 0.33	0.695	0.32 ± 0.45	0.485

*Note*: Data show the beta coefficient (β) and standard error (SE) of the interaction effect of supplementation (vehicle vs. CSE) and group (control vs. MUN).

Abbreviations: CSE, cocoa shell extract; MUN, maternal undernutrition.

MUN‐VEH males and females had significantly higher SBP compared with C‐VEH sex‐matched counterparts. C‐CSE male and female rats did not show differences in blood pressure compared with C‐VEH. MUN‐CSE male and female rats had significantly lower SBP compared with MUN‐VEH sex‐matched counterparts (Table 1). A significant interaction was detected between supplementation × group for both sexes (Table 2).

### 3.2 Heart structure and function

**FIGURE 2:**
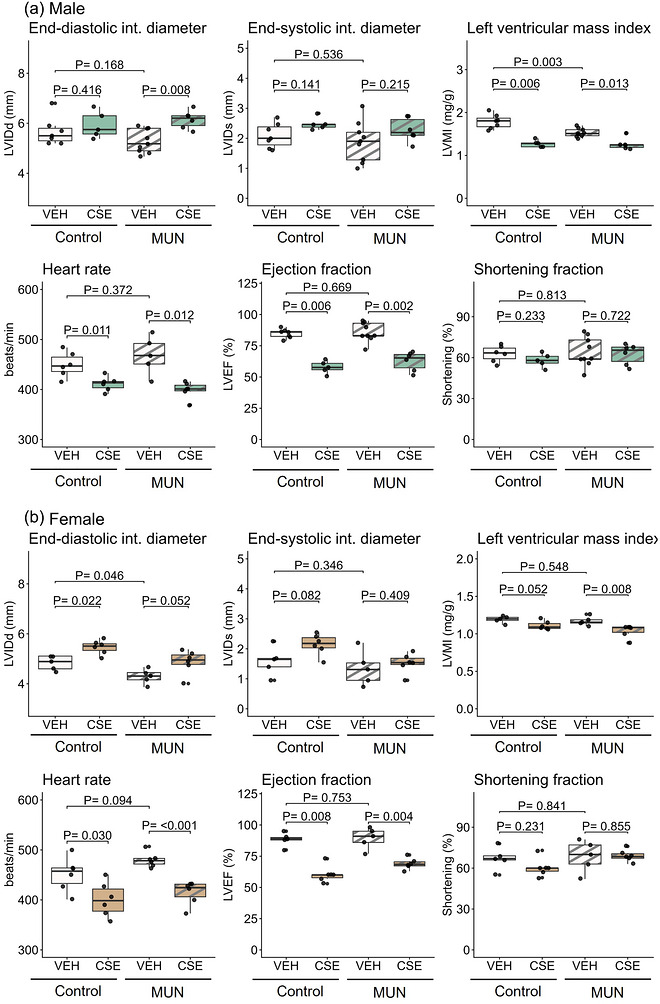
Echocardiographic data from males (a) and females (b). Data show the median and interquartile range [Q1; Q3] and *P*‐values (P) extracted from the Mann–Whitney *U*‐test; *n* = 6–8 animals per group, shown as individual data points. Abbreviations: CSE, cocoa shell extract; LVEF, left ventricular ejection fraction; LVIDd, left ventricular internal (int.) diameter at diastole; LVIDs, left ventricular internal diameter at systole; LVMI, left ventricular mass index; MUN, maternal undernutrition; VEH, vehicle.

In male rats, LVMI was significantly smaller in MUN‐VEH compared with C‐VEH, whereas LVIDd, LVIDs, ejection fraction, heart rate and fractional shortening were not significantly different between groups. C‐CSE males had significantly lower LVMI, heart rate and ejection fraction compared with C‐VEH, and no changes were observed in fractional shortening. MUN‐CSE males also had significantly smaller LVMI, heart rate and ejection fraction, whereas LVIDd was larger compared with MUN‐VEH males, and no changes were observed in fractional shortening (Figure 2a). In male rats, a significant interaction was detected only for LVMI (Table 2). In females, LVIDd was significantly larger in MUN‐VEH compared with C‐VEH, without significant differences in the other parameters. C‐CSE females had significantly larger LVIDd and lower LVMI, heart rate and ejection fraction, without modification in fractional shortening. Similar findings were reported for MUN‐CSE females: larger LVIDd and reduced LVMI, heart rate and ejection fraction (Figure 2b). In female rats, no significant interaction between supplementation × group was detected for any of the evaluated parameters (Table 2).

### 3.3 Resistance artery function

In male rats, no significant differences were observed between MUN‐VEH and C‐VEH in MRA responses to KCl, NA maximal responses or pD_2_ values. In C‐CSE males and MUN‐CSE males, no significant differences in KCl or NA maximal contractions were detected compared with VEH counterparts (Table 3). No interaction was detected in these parameters (Table 2).

**TABLE 3:** Maximum response and pD_2_ values to the different vasoactive agonists.

Parameter	Control		*P* ^1^	MUN		*P* ^2^	*P* ^model^
**Male**	VEH (*n* = 10)	CSE (*n* = 5)		VEH (*n* = 9)	CSE (*n* = 9)		
K^+^ (Max.)	25.3 [23.2; 29.5]	21.2 [20.9; 21.4]	0.107	25.6 [21.1; 28.3]	22.5 [16.6; 24.3]	0.085	0.665
NA (Max.)	120 [115; 122]	120 [116; 124]	0.713	122 [119; 132]	119 [117; 120]	0.122	0.236
NA (pD_2_)	5.63 [5.62; 5.66]	5.67 [5.60; 5.77]	0.624	5.68 [5.54; 5.76]	5.73 [5.65;5.74]	0.566	0.414
ACh (Max.)	77.6 [75.8; 86.5]	43.3 [38.4; 47.0]	**0.005**	61.4 [51.1; 73.4]	69.9 [65.9; 75.1]	0.563	**0.021**
ACh (pD_2_)	7.62 [7.33; 7.86]	7.20 [7.15; 7.25]	**0.034**	7.18 [7.11; 7.40]	7.35 [7.16; 7.48]	0.817	0.062
SNP (Max.)	60.7 [57.1; 66.9]	54.7 [53.8; 55.6]	0.242	57.5 [55.1; 70.2]	55.0 [49.9; 58.5]	0.366	0.775
SNP (pD_2_)	7.45 [6.89; 7.61]	6.91 [6.85; 6.96]	0.380	6.67 [6.25; 6.94]	6.75 [6.35; 7.48]	0.366	**0.046**
**Female**	VEH (*n* = 10)	CSE (*n* = 5)		VEH (*n* = 6)	CSE (*n* = 7)		
K^+^ (Max.)	23.4 [20.9; 25.8]	16.3 [14.9; 18.4]	**0.010**	27.1 [22.1; 29.8]	18.8 [18.0; 20.6]	**0.046**	0.329
NA (Max.)	120 [111; 122]	116 [110; 119]	0.841	126 [125; 129]	120 [109; 121]	**0.015**	**0.009**
NA (pD_2_)	5.64 [5.62; 5.73]	5.59 [5.42; 5.70]	0.257	5.69 [5.64; 5.73]	5.69 [5.69; 5.89]	0.317	0.906
ACh (Max.)	88.9 [85.6; 97.0]	48.6 [42.4; 53.6]	**0.008**	98.0 [97.4; 99.2]	60.5 [58.5; 66.0]	**0.004**	**0.046**
ACh (pD_2_)	7.42 [7.30; 7.69]	7.12 [6.94; 7.35]	0.257	7.55 [7.48; 7.63]	7.32 [7.12; 7.38]	0.109	0.391
SNP (Max.)	70.0 [68.2; 72.4]	46.8 [43.8; 49.9]	**0.040**	73.2 [62.7; 76.2]	57.1 [53.0; 65.8]	**0.045**	0.775
SNP (pD_2_)	6.69 [6.51; 6.97]	6.49 [6.45; 6.53]	0.242	7.14 [7.12; 7.55]	6.43 [6.33; 7.05]	0.068	0.086

*Note*: Data show the median and interquartile range [Q1; Q3]. The *P*‐value was extracted from the Mann–Whitney *U*‐test, with *P* ^1^ being control‐VEH vs. control‐CSE; *P* ^2^, MUN‐VEH vs. MUN‐CSE; and *P* ^model^, control vs. MUN in vehicle (VEH) groups. Significant differences are highlighted in bold.

Abbreviations: ACh, acetylcholine; CSE, cocoa shell extract; Max, maximum response; MUN, maternal undernutrition; NA, noradrenaline; pD_2_, −log_10_[EC_50_]; SNP, sodium nitroprusside; VEH, vehicle.

**TABLE 4:** Summary of the main results.

Parameter		MUN model	Effects of CSE during lactation in	
			Control	MUN
Weight Blood pressure	Weight	♂♀↓	♂♀↓	♂↓
	Blood pressure	♂♀↑		♂♀↓
Left ventricular structure and function	End‐diastolic internal diameter	♀↓	♀↑	♂♀↑
	Indexed mass	♂↓	♂♀↓	♂♀↓
	Ejection fraction		♂♀↓	♂♀↓
	Heart rate		♂♀↓	♂♀↓
MRA function	Vasoconstriction		♀↓	♀↓
	Vasodilatation (endothelium dependent)	♂↓	♂♀↓	♀↓
	Vasodilatation (endothelium independent)	♂↓		
MRA mechanical and structural properties	Internal diameter	♂♀↓		♂♀↑
	Cross‐sectional area			♂♀↑
	Wall‐to‐lumen ratio		♀♂↑	
	Stiffness	♂↑		
MRA cell density	Adventitial thickness	♂↑		
	Medial thickness	♂↓	♂♀↑	♂♀↑
	Smooth muscle cells		♂♀↓	♂↓

Abbreviations: CSE, cocoa shell extract; MRA, mesenteric resistance artery; MUN, maternal undernutrition; ♂, male; ♀, female; ↓, decrease; ↑, increase.

In females, maximal responses to NA were higher in MUN‐VEH compared with C‐VEH.

C‐CSE females and MUN‐CSE females had significantly lower KCl responses compared with respective VEH counterparts. No interaction between supplementation × group was detected. C‐CSE females did not shown significant differences in maximal NA responses or pD_2_. However, MUN‐CSE females had lower NA maximal responses compared with MUN‐VEH (Table 3). A significant interaction between supplementation × group was detected in females (Table 2).

**FIGURE 3:**
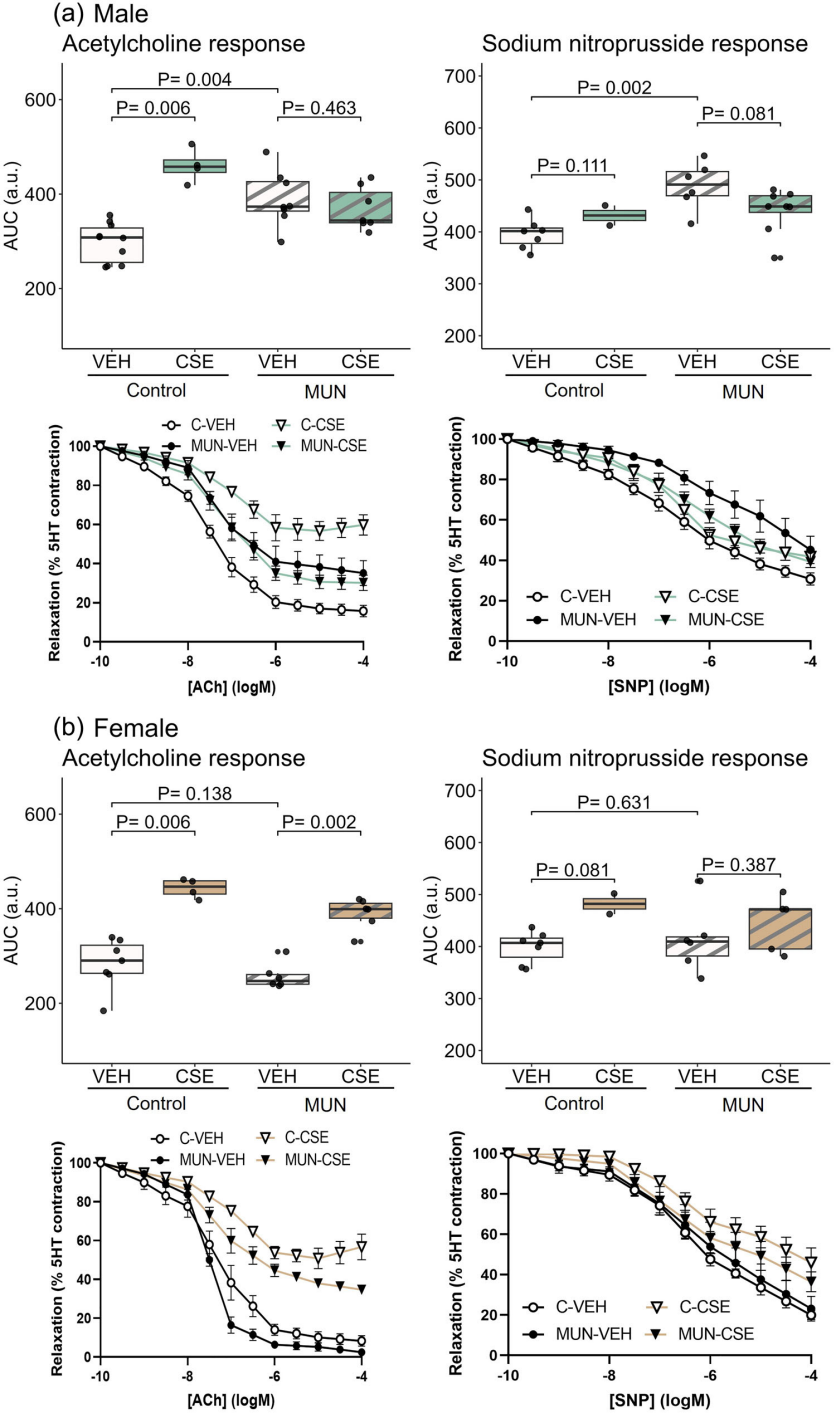
Effect of vasodilators in mesenteric resistance arteries from males (a) and females (b). Top graphs show the median and interquartile range [Q1; Q3] of the area under the curve, with *P*‐values (P) being extracted from the Mann–Whitney *U*‐test; *n* = 5–8 animals per group, shown as individual data points. Bottom graphs show the concentration–response curves. Abbreviations: ACh, acetylcholine; AUC, area under the curve; 5‐HT, serotonin; CSE, cocoa shell extract; MUN, maternal undernutrition; SNP, sodium nitroprusside; VEH, vehicle.

Regarding vasodilatory responses, male rats MUN‐VEH had significantly larger ACh and SNP AUCs compared with C‐VEH, being pD_2_ values to SNP not significantly different. In C‐CSE males, ACh AUC was significantly larger compared with C‐VEH, and maximal responses were significnatly reduced. No differences between groups were detected in SNP responses. In MUN‐CSE males, ACh AUC or maximal responses were not different compared with MUN‐VEH counterparts (Figure 3a; Table 3). A significant interaction between supplementation × group was detected for ACh, but not for SNP (Table 2).

In females, no significant differences in vasodilator responses were found between MUN‐VEH and C‐VEH rats. In C‐CSE females, the AUC for ACh was significantly larger and maximal responses to both ACh and SNP significantly reduced compared with C‐VEH females (Figure 3a; Table 3). In MUN‐CSE females, ACh AUC was larger, and maximal responses to both ACh and SNP were reduced compared with MUN‐VEH (Figure 3b; Table 3). No significant interaction between supplementation × group was detected for ACh or SNP in females (Table 2).

### 3.4 Resistance artery structure and mechanics

**FIGURE 4:**
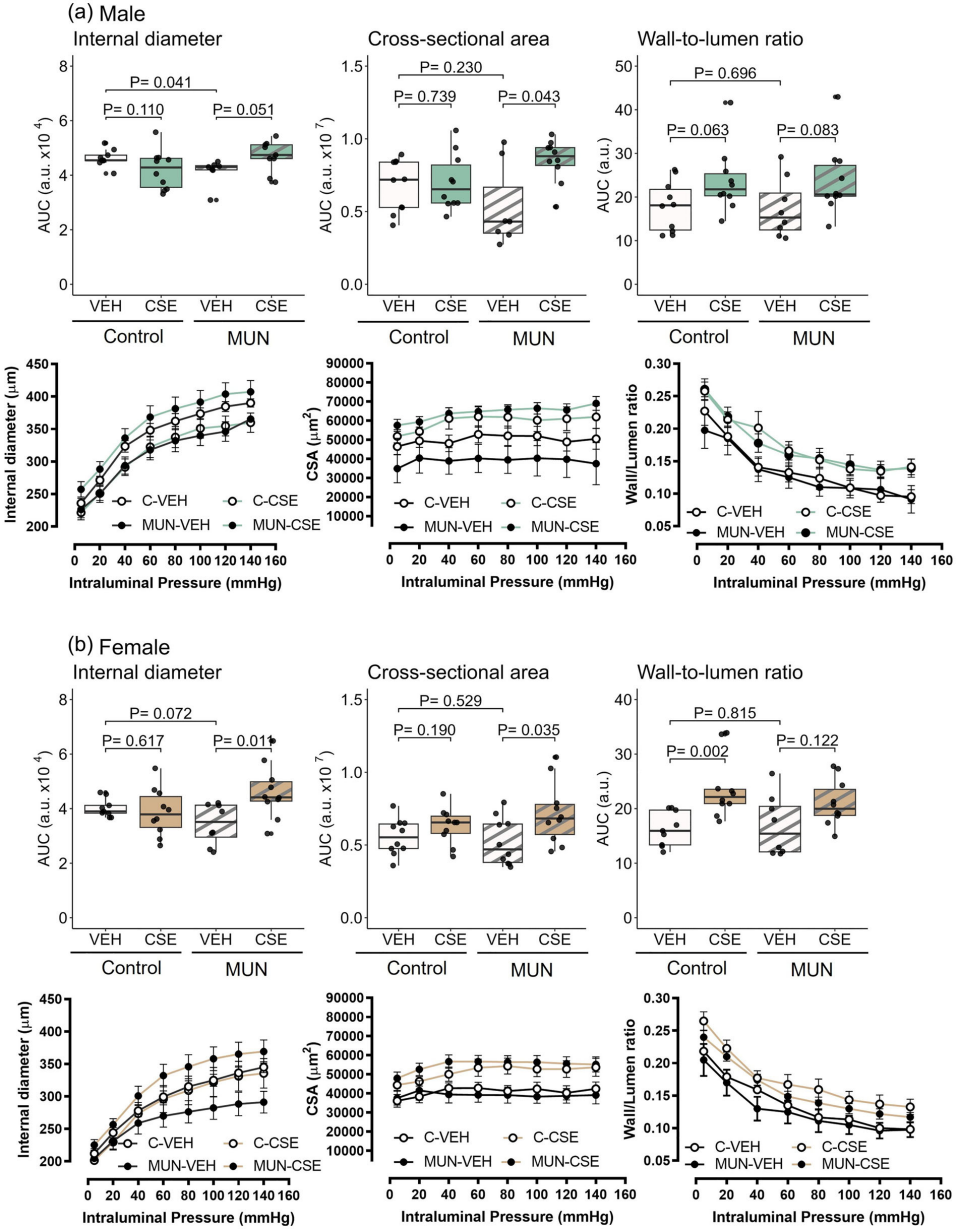
Gross structure of mesenteric resistance arteries from males (a) and females (b). Top graphs show the median and interquartile range [Q1; Q3] of the area under the curve, with *P*‐values (P) being extracted from the Mann–Whitney *U*‐test; *n* = 8–10 animals per group, shown as individual data points. Bottom graphs show the pressure curves. Abbreviations: AUC, area under the curve; CSA, cross‐sectional area; CSE, cocoa shell extract; MUN, maternal undernutrition; VEH, vehicle.

In male rats, MUN‐VEH had significantly smaller internal diameter compared with C‐VEH. No differences were observed between C‐CSE compared with C‐VEH. MUN‐CSE males had significantly larger internal diameter compared with non‐supplemented MUN‐VEH counterparts. CSA was not significantly different in MUN‐VEH compared with C‐VEH control males. In control males, supplementation did not modify CSA, which was not significantly different between C‐CSE and C‐VEH. However, supplementation in MUN males increased CSA, with this parameter being significantly larger in MUN‐CSE compared with non‐supplemented MUN‐VEH counterparts. Regarding wall‐to‐lumen ratio, no significant differences between C‐VEH and MUN‐VEH groups were observed. Supplementation tended to increase this parameter in C‐CSE males, without statistical differences, and did not modify wall‐to‐lumen ratio in MUN‐CSE compared MUN‐VEH males (Figure 4a). In males, a significant interaction between supplementation × group was detected for internal diameter and CSA, but not for wall‐to‐lumen ratio (Table 2).

In females, MUN‐VEH rats had a smaller internal diameter compared with C‐VEH, but this did not reach statistical significance. CSE supplementation did not influence internal diameter in control females, with no significant differences between C‐CSE and C‐VEH groups. Instead, MUN‐CSE internal diameter was significantly larger compared with MUN‐VEH females. No significant difference was detected in CSA between C‐VEH and MUN‐VEH females. In control females, CSE supplementation did not influence CSA. However, CSA was significantly larger in MUN‐CSE compared with MUN‐VEH. The wall‐to‐lumen ratio was larger in C‐CSE compared with C‐VEH, and no difference was detected between MUN‐CSE and MUN‐VEH females (Figure 4b). In females, a significant interaction between supplementation × group was detected for internal diameter but not for CSA and wall‐to‐lumen ratio (Table 2).

Mechanical function was evaluated through the β‐value, a parameter that is inversely proportional to elasticity. MUN‐VEH males had significantly larger β‐values (8.01 [7.13; 8.82] a.u.) compared with control C‐VEH counterparts (6.13 [5.10; 6.73] a.u.; *P* = 0.039), which suggest vascular stiffness. CSE supplementation in male control rats did not modify this parameter, with no statistical difference between C‐CSE (5.64 [4.45; 6.38]) and C‐VEH (6.13 [5.10; 6.73]). MUN‐CSE rats had a lower β‐value (5.74 [4.35; 7.18] a.u.) compared with MUN‐VEH, but this did not reach statistical significance (8.01 [7.13; 8.82] a.u.; *P* = 0.065).

In females, no significant differences in β‐values were detected between C‐VEH (5.43 [4.43; 6.62] a.u.) and MUN‐VEH (6.81 [5.03; 8.20] a.u.; *P* = 0.226). No significant differences were found between C‐VEH (5.43 [4.43; 6.62] a.u.) and C‐CSE females (6.03 [4.65; 7.04] a.u.) or between MUN‐VEH (6.81 [5.03; 8.20] a.u.) and MUN‐CSE (5.65 [4.34; 7.42] a.u.; *P* = 0.545). No significant interaction between supplementation × group was detected for β‐values either in males or in females (Table 2).

**FIGURE 5:**
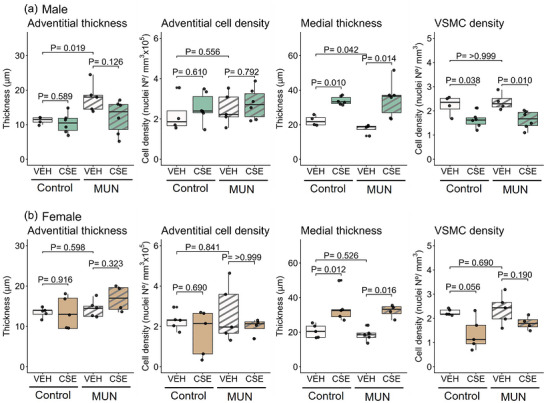
Medial and adventitial layer in mesenteric resistance arteries from males (a) and females (b). Graphs show the median and interquartile range [Q1; Q3] and *P*‐values (P) extracted from the Mann–Whitney *U*‐test; *n* = 5–6 animals per group, shown as individual data points. Abbreviations: CSE, cocoa shell extract; MUN, maternal undernutrition; VEH, vehicle; VSMC, vascular smooth muscle cell.

Possible changes in medial and adventitial layers were analysed further with confocal microscopy. MUN‐VEH males had significantly larger adventitial layer thickness, without changes in cell density compared with C‐VEH. CSE supplementation did not influence adventitial parameters, in either control or MUN males. Media thickness was significantly smaller in MUN‐VEH compared with C‐VEH males, without modifications in VSMC density. CSE supplementation enlarged media thickness in both C‐CSE and MUN‐CSE compared with their respective non‐supplemented counterparts. VSMC density was significantly smaller in C‐CSE and MUN‐CSE compared with their non‐supplemented counterparts (Figure 5a). In male rats, no significant interaction between supplementation × group was detected for adventitial and medial layer parameters (Table 2).

In females, no significant differences in the adventitial layer were detected between MUN‐VEH and C‐VEH groups, either in thickness or in cell density. CSE supplementation did not influence adventitial parameters. Medial layer thickness and density were not different between MUN‐VEH and C‐VEH females. C‐CSE females had significantly larger media thickness and tended to have lower VSMC density (*P* = 0.056). MUN‐CSE females had larger thickness, without significant changes in density compared with MUN‐VEH. In female rats, no significant interaction between supplementation × group was detected for adventitial or medial layer parameters (Table 2).

## 4 DISCUSSION

Cocoa shell is one of the main by‐products derived from the chocolate industry, rich in protein, dietary fibre and bioactive compounds, with wide applications, including health, which could contribute to solve the environmental issue of its disposal (Panak Balentić et al., 2018). Our group has previously obtained an aqueous extract (CSE), with the main components being theobromine, caffeine and phenolic compounds, mainly gallic and protocatechuic acids and flavan‐3‐ols, which resist gastrointestinal digestion (Panak Balentić et al., 2018). In vitro, CSE has important antioxidant and anti‐inflammatory properties in cells (Cañas et al., 2023; Rebollo‐Hernanz et al., 2019) and vasodilatory actions in several arteries through superoxide anion scavenging activity of its main components (Rodríguez‐Rodríguez et al., 2022b). We have also demonstrated that CSE supplementation in adult MUN rats with established hypertension reduced blood pressure and improved endothelial dysfunction (Ruvira et al., 2024). In the context of DOHaD, pregnancy and lactation are target windows for reprogramming, and interventions with antioxidants during these periods have demonstrated efficacy to prevent the development of hypertension in the offspring (Tain & Hsu, 2022), although adverse effects have also been reported in pregnancy. Most studies analyse supplementation during this period, and there is a scarcity of data exploring lactation exclusively. This prompted the present study, particularly given that we have evidence that this period consolidates a pro‐hypertensive phenotype in the MUN model (Monedero Cobeta et al., 2024; Rodríguez‐Rodríguez et al., 2022a). The main conclusions of our study are that CSE administered during lactation provided little benefit to MUN rats and, overall, was not beneficial, in contrast to our previous reported effects with CSE supplementation in adulthood. Although it prevented the development of hypertension in MUN rats, it induced profound alterations in the heart and resistance vasculature, particularly in non‐programmed offspring, which might be detrimental.

CSE supplemented through lactation reduced body growth in most groups. We do not think there was an alteration in maternal care, because maternal behaviour was normal in all groups. Instead, we think this is related to the bioactive components of CSE. Based on our previous findings on the impact of lactation for adipocyte growth (Rodríguez‐Rodríguez et al., 2022a), we suggest that adipose tissue might be one of the tissues modified. In adult mice with diet‐induced‐obesity, we have previously demonstrated that CSE supplementation at 500 mg/kg/day reduced white adipocyte size and increased browning (Braojos et al., 2023). This effect has been reported for the main CSE components, theobromine, polyphenols and caffeine. Theobromine can enhance conversion of white to beige adipocytes (Tanaka et al., 2022). CSE phenolics promote browning in cultured adipocytes (Rebollo‐Hernanz et al., 2019), and a high flavan‐3‐ol extract administered through lactation in obese dams improved the inflammatory profile in white adipose tissue of offspring (Mariné‐Casadó et al., 2022). Caffeine consumption reduced body weight gain in mice, inhibiting fat accumulation (Kong et al., 2021). In the present study and in our prior studies, we did not evaluate food intake of the animal. It has been demonstrated that administration of caffeine during lactation induced a reduction of cerebellum weight without a modification in food consumption (Yazdani et al., 2004). However, the effect of CSE or some of its components on appetite in our experimental model deserves attention. The question of whether CSE bioactive components reach maternal milk also remains. Although we do not have direct evidence, we think it is possible, based on our metabolomic study, which demonstrated the presence of caffeine and theobromine in rat plasma after 1 week of CSE supplementation (Ramiro‐Cortijo et al., 2025), and the work of Yazdani et al. (2004), who evidenced caffeine in rat breastmilk after maternal supplementation. It should be noted that both studies demonstrated that supplementation induced alterations glycerophospholipids and sphingosines, lipid signalling molecules that might affect both growth and development.

At the age of 8–10 months, SBP was elevated in MUN males and females. We have previously described that female MUN rats are still normotensive by the age of 5–6 months (Rodríguez‐Rodríguez et al., 2017), but show high blood pressure by the age of 18 months (Gutiérrez‐Arzapalo et al., 2020), supporting their better adaptation to environmental insults, as commonly described in DOHaD studies (Morton et al., 2016). The present data indicate that hypertension in MUN females develops earlier than expected. CSE supplementation had different actions on blood pressure depending on the group, with a lowering effect on MUN rats and no effects on control offspring. Blood pressure is determined by cardiac output and peripheral vascular resistance. CSE supplementation produced heart alterations consistent with reduced cardiac output across all groups, an effect that would be expected to lower blood pressure. However, CSE induced divergent changes in the resistance vasculature of MUN and control rats, which can explain the differential effect on blood pressure. CSE‐supplemented MUN rats maintained or reduced vascular tone and stiffness, whereas supplementation in control rats reduced vasodilatation and enhanced vascular resistance.

CSE supplementation during lactation induced an important structural alteration in the heart, irrespective of group and sex. This effect confirms the importance of postnatal life for the development of the phenotype initiated by a developmental insult, evidenced in experimental models of uteroplacental insufficiency (Chou & Chen, 2023) and maternal undernutrition (Rodríguez‐Rodríguez et al., 2021) and in children exposed to fetal growth restriction (Crispi et al., 2010). Lactation is a period when cardiac morphogenesis continues in the rat (Zicha & Kuneš, 1999), and therefore, it can shape the phenotype initiated by a uterine insult. Our results suggest that the reduced ventricular mass and ejection fraction in adult offspring supplemented with CSE in lactation could contribute to the observed blood pressure‐lowering effects. Our data are in accordance with other DOHaD models. For example, animals exposed to fetal hypoxia and supplement with melatonin exhibit to a decrease in cardiomyocyte density (Paz et al., 2026). There was reduced ejection fraction in mice supplemented during fetal life with high doses of folic acid (Cai et al., 2024). Polyphenols inactivate cardiac hypertrophic factors, such as angiotensin II (Hedayati et al., 2023). Supplementation of mice with tea extracts rich in flavan‐3‐ols and xanthine attenuated angiotensin II‐induced cardiac hypertrophy (de la Fuente‐Muñoz et al., 2025), and pressure overload‐induced cardiac hypertrophy in mice was reduced by cocoa polyphenols (Sari et al., 2020). Given the role of angiotensin II in DOHaD models (Morton et al., 2016) and, particularly, in the development of cardiac hypertrophy in MUN rats during lactation (Monedero Cobeta et al., 2024; Rodríguez‐Rodríguez et al., 2021), we suggest that CSE bioactive components might interfere with the renin–angiotensin system, with the long‐term consequence of a reduction of cardiac mass. The cardiac hypotrophic effect of CSE might also be a consequence of compounds derived from changes in maternal metabolism. According to our recent metabolomic study in control female rats, CSE altered lipid signalling processes that are crucial in cell growth and differentiation (Ramiro‐Cortijo et al., 2025), including postnatal cardiomyocyte development (Ji et al., 2024).

CSE also induced a reduction in heart rate, which would contribute to a lower cardiac output. We do not think that this effect is related to the methylxanthines present in the extract, which, at least in humans, induce a stimulating effect on fetal heart rate (Buscicchio et al., 2012). There is evidence of the effectiveness of some antioxidants to treat ventricular tachycardia (Szyller et al., 2022) and, in rats, resveratrol reduces cardiac muscle contraction and action potential duration, associated with an alteration in K_ATP_ channels (Buluc et al., 2007). However, scientific literature on the influence of polyphenols on cardiac electrical activity is scarce, and to the best of our knowledge, there is no information about its effects during early periods of life, an aspect that deserves further attention.

Blood pressure is also controlled by vascular peripheral resistance, largely dependent on the function and structure of resistance vessels. Therefore, we assessed possible modifications induced by CSE during lactation using the MRA, which is a representative resistance artery. MUN rats exhibited endothelial dysfunction in males, increased NA contractions in females, and inward remodelling in both, alterations commonly found in models of developmental hypertension (Hsu & Tain, 2021; Morton et al., 2016), which can explain blood pressure elevation though increased peripheral resistance. CSE supplementation during lactation worsened endothelium‐dependent responses in groups with prior normal vasodilatation and did not substantially modify responses in MUN males that exhibited endothelial dysfunction. This finding was surprising, given our previous results for adult rat supplementation with the same CSE dose and duration, evidencing restoration of vasodilatory responses in MUN rats without deleterious consequences in control animals (Ruvira et al., 2024). These data suggest interference of CSE components with the growing endothelium, which in rat MRA develops fully in the postnatal period. Although we did not evaluate this in the present work (and thus this remains speculative), literature data support this assumption. First, in rat mesenteric vessels, endothelium‐dependent dilatation in response to acetylcholine is gradually increased during lactation owing to significant changes in the expression of some channels and enzymes responsible for synthesis of vasodilator factors (Nourian et al., 2023). Second, several polyphenols, including catechins, reduce endothelial cell proliferation and angiogenesis (Gallemit et al., 2021; Oak et al., 2005; Rashidi et al., 2017), with beneficial outcomes in reducing tumour progression, but which could be detrimental in a developing rat. In fact, we suggest that a reduction in endothelial vasodilators, particularly nitric oxide, during early life could participate in the observed medial layer hypertrophy induced by supplementation. The trophic response is likely to involve VSMC enlargement, because VSMC density was not modified, which is compatible with a reduction of NO‐dependent inhibition of growth‐promoting signalling pathways (Kapakos et al., 2010). We discarded an increase in extracellular matrix (ECM) in the medial enlargement, because arterial stiffness did not increase with CSE supplementation.

Although we cannot rule out a direct effect of CSE components on VSMC hypertrophy, the available data do not support this assumption, because polyphenols given during adult life inhibit VSMC phenotypic switching towards a hypertrophic secretory phenotype (Du et al., 2025; Serino & Salazar, 2018; Shohrati et al., 2025).

Despite similar VSMC hypertrophy induced by CSE supplementation in all groups, there were important structural differences in control and MUN rats. In MUN males and females, medial enlargement was accompanied by a diameter expansion, whereas in C‐CSE males and females, lumen expansion did not occur, resulting in increased wall‐to‐lumen ratio. These changes would increase peripheral resistance, with deleterious consequences, because an elevated wall‐to‐lumen ratio is a marker of cardiovascular risk (Rizzoni & Rosei, 2006). This dual response might explain the blood pressure reduction in MUN but not control supplemented rats.

A relevant issue is the CSE dose used. We gave 250/mg/kg day based on our previous positive results in adult rats (Ruvira et al., 2024) and aged rats (Ruvira et al., 2023). It is possible that this dose was adequate in an adult organism but excessive in an immature rat. This assumption is supported by the study by Petit et al. (2016), who revealed that resveratrol administration for 2 weeks at 37.5 mg/kg/day induced MRA hypertrophy, whereas this effect was not observed at 5 mg/kg/day. This dual effect of resveratrol has also been evidenced in other rodent models of disease, such as insulin resistance (Baron et al., 2014).

### 4.1 Limitations and future studies

The present study demonstrates that CSE supplementation during lactation induces important cardiac and vascular changes that could be detrimental overall, particularly in healthy control offspring. These results raise important questions that were not addressed in the present work and ask for further studies. The first is the dosage; we tested a only single dose, which was adequate for adult rats but might be excessive during development. Therefore, future studies must determine an adequate dose for the neonatal period. Secondly, it would be interesting to evaluate what mechanisms are implicated in the complex responses induced by CSE supplementation in cardiac and vascular tissues to understand the long‐term consequences. Thirdly, it would be important to evaluate whether the observed modifications are related to changes in maternal behaviour, milk production or milk composition. Finally, it would be important to gain information on the role of specific phytochemicals in the extract, particularly the bioactive components with the highest concentrations: caffeine, theobromine and catechins.

## 5 CONCLUSIONS

The present study demonstrates that CSE supplementation during lactation induces long‐term structural and functional cardiovascular alterations that, overall, might have deleterious effects, particularly in healthy offspring. Our findings suggest that the dose used (which was effective in counteracting the consequences of fetal programming administered during adult life) might be excessive during developmental periods. These results raise important concerns regarding the safety of supplementation during the lactation period and highlight the need for further research to achieve a comprehensive understanding of the physiological effects of polyphenol and methylxanthine intake, and the adequate doses, ultimately contributing to an improvement in safety. The summary of results is shown in Table 4.

## AUTHOR CONTRIBUTIONS

Conceptualization: Silvia M. Arribas. Methodology: Santiago Ruvira, Pilar Rodríguez‐Rodríguez, Metee Iampanichakul, Begoña Quintana‐Villamandos and Dolores Morales. Formal analysis and investigation: Begoña Quintana‐Villamandos and David Ramiro‐Cortijo. Writing—original draft preparation: Santiago Ruvira, Pilar Rodríguez‐Rodríguez and Metee Iampanichakul. Writing—review and editing: David Ramiro‐Cortijo and Silvia M. Arribas. Funding acquisition: Silvia M. Arribas. Resources: Begoña Quintana‐Villamandos, Dolores Morales and Silvia M. Arribas. Supervision: David Ramiro‐Cortijo and Silvia M. Arribas. All authors approved the final version of the manuscript and agree to be accountable for all aspects of the work in ensuring that questions related to the accuracy or integrity of any part of the work are appropriately investigated and resolved. All persons designated as authors qualify for authorship, and all those who qualify for authorship are listed.

## CONFLICT OF INTEREST

The authors declare no conflict of interest. The funders had no role in the design of the study; in the collection, analyses, or interpretation of data; in the writing of the manuscript; or in the decision to publish the results.

## GENERATIVE AI STATEMENT

The authors declare that AI has not been used in any stage of the research and/or writing process of this manuscript.

## Data Availability

The corresponding author had full access to all the data in the study and takes responsibility for the integrity of the data and the accuracy of the data analysis. The raw data supporting the conclusions of this article will be made available by the authors on request and sending will be evaluated following Spanish ethical regulations.
